# Isolation of Multidrug-Resistant *Mycobacterium Avium* Subsp. *Avium* from a Wild Eurasian Otter (Lutra Lutra)

**DOI:** 10.3390/antibiotics13070591

**Published:** 2024-06-26

**Authors:** Irena Reil, Sanja Duvnjak, Silvio Špičić, Gordan Kompes, Antonela Bagarić, Martina Đuras, Andrea Gudan Kurilj, Maja Lukač, Mišel Jelić, Maja Zdelar-Tuk

**Affiliations:** 1 Croatian Veterinary Institute, Savska Cesta 143, 10000 Zagreb, Croatia; reil@veinst.hr (I.R.); spicic@veinst.hr (S.Š.); kompes@veinst.hr (G.K.); marijan@veinst.hr (A.B.); zdelar-tuk@veinst.hr (M.Z.-T.); 2 Faculty of Veterinary Medicine, University of Zagreb, Heinzelova 55, 10000 Zagreb, Croatia; martina.duras@vef.unizg.hr (M.Đ.); agudan@vef.unizg.hr (A.G.K.); mlukac@vef.hr (M.L.); 3 Varaždin City Museum, Department of Natural Sciences, Šetalište Josipa Jurja Strossmayera 1, 42000 Varaždin, Croatia; misel.jelic@gmv.hr

**Keywords:** *Mycobacterium avium* subsp. *avium*, otters, antimicrobial resistance, antibiotics, zoonoses

## Abstract

*Mycobacterium avium* subsp. *avium* is pathogenic mainly to birds, although cases of mycobacteriosis caused by these bacteria have also been reported in other animals and humans. Not much is known about the effects of this pathogen on otters. The aim of this study was to report for the first time the isolation of *M. avium* subsp. *avium* in wild otter and to describe its multidrug resistance profile. A female otter injured in a car accident was found dead and subjected to postmortem examination. Apart from the trauma changes, no other macroscopic pathological changes were detected. Bacteriologic examination revealed the presence of acid-fast bacilli in the lymph nodes, which were confirmed by molecular methods as *M. avium* subsp. *avium*. Antimicrobial susceptibility testing revealed susceptibility to clarithromycin and amikacin, but resistance to linezolid, moxifloxacin, streptomycin, isoniazid, trimethoprim/sulfamethoxazole, ciprofloxacin, doxycycline, and ethionamide. This is unusual for wild species, which generally should not come into contact with antimicrobials, and may suggest that multidrug-resistant MAC strains are circulating between wild and domestic animals. These results emphasise the need for additional epidemiological studies on non-tuberculous mycobacteria in wildlife and their implications for one health.

## 1. Introduction

The genus *Mycobacterium* comprises a group of ubiquitous organisms with more than 270 described species, ranging from harmless saprophytes to significant pathogens [1]. In addition to the species that cause tuberculosis (*Mycobacterium tuberculosis* complex), there are also species known as non-tuberculous mycobacteria (NTM) which generally have low pathogenicity for humans but can cause a variety of clinical diseases [2], with *Mycobacterium avium* complex (MAC) being the most frequently isolated NTM in the European Union [3]. The complex originally consisted of two species, *M. avium* and *M. intracellulare* [4]. The most clinically important subspecies in animals and humans within *M. avium* are *M. avium* subsp. *avium* (MAA), *M. avium* subsp. *silvaticum* (MAS), *M. avium* subsp. *paratuberculosis* (MAP), and *M. avium* subsp. *hominissuis* (MAH), each of which has specific characteristics in terms of pathogenicity and host spectrum [5]. Despite their taxonomic relationship, these subspecies are phenotypically distinct organisms, ranging from environmental bacteria that cause opportunistic infections to those that cause severe infections in ruminants, swine, and birds (poultry and pet parrots), such as paratuberculosis, avian tuberculosis, and other mycobacteriosis. MAC are widely distributed in the environment and have been isolated from soil, water, aerosols, protozoa, and plants [6]. 

Although traditionally considered non-pathogenic, they can cause severe forms of disease and destructive tissue lesions even in immunocompetent individuals [7]. The most commonly found NTM pathogen in humans is MAH; however, less frequently, infections have also been linked to MAA [8,9]. In humans, MAA can cause lymphadenitis, pulmonary disease, and a disseminated form of infection, especially in immunocompromised individuals [10,11,12]. Although MAA has been isolated from a variety of animal species, including pigs, goats, sheep, and cattle, it is considered highly host-specific and a strict pathogen for birds [13,14,15]. Wild birds have long been considered the main reservoir of MAA, responsible for its release and spread in the environment. However, the diversity of strain types suggests that infections are not acquired from a single reservoir and, with this in mind, further studies are needed to identify the avian reservoirs and environmental sources of MAA [16]. Recent and regional studies described MAA isolates in domestic pigs, wild boar, cattle, chickens, and domestic ducks in Croatia [17,18,19,20]. MAA has also been associated with infections in badger, buzzard, wild goat, rabbit, fox, American mink, red deer, roe deer, stone marten, wild boar, domestic pigs, poultry, and cattle in Slovenia [14] and Spain where MAA was the species most frequently isolated from animals showing lesions compatible with mycobacteriosis [21]. To our knowledge, there are no reports of MAA infection in Eurasian otters to date. However, there are reported cases of infection with *M. bovis*, *M. microti,* and MAP in Eurasian otters in Europe, more precisely Northern Ireland, France, and Portugal [22,23,24]. The animals infected with *M. bovis* and *M. microti* showed tuberculosis-like lesions, while MAP was detected by culture and PCR methods without typical lesions. 

The drugs used to treat MAC infections in humans are macrolides, clofazimine, rifampin, rifabutin, ethambutol, fluoroquinolones, linezolid, and aminoglycosides [25]. Due to the risk of developing macrolide resistance [26,27,28,29], in more severe cases, a fourth agent, such as injectable streptomycin or amikacin, is added [27]. The second-line drugs used when resistant strains emerge that have not responded to the first-line drugs (clarithromycin and amikacin), as well as in patients who cannot tolerate macrolide therapy, are linezolid and moxifloxacin [28,29]. The treatment of mycobacterial diseases in veterinary medicine is controversial, as there are no approved drugs for animals and there is a potential zoonotic risk. In addition, MAC responds poorly to treatment. If treatment is chosen, drugs used for mycobacterial infections in humans should be used. The described treatment in cats and dogs is long-term, starting with an initial phase of two to three drugs (rifampicin–fluoroquinolone–clarithromycin/azithromycin) for two months and continuing with two drugs (rifampicin and either a fluoroquinolone or clarithromycin/azithromycin) for a further four to six months. As rifampicin is often poorly tolerated and may have to be discontinued early, doxycycline is used instead [30,31]. The treatment of systemic mycobacteriosis is generally not effective and is not recommended in dogs [32,33]. The treatment of MAA infections in poultry is not recommended as it is unlikely to be successful due to its duration and cost [16]. The treatment of pet birds with MAA infection is described using isoniazid, rifampin, rifabutin, ethambutol, clofazimine, ciprofloxacin, enrofloxacin, streptomycin, and amikacin. The initial treatment regimen should include rifabutin and ethambutol, and azithromycin or clarithromycin may be given later, with the addition of fluoroquinolones or an aminoglycoside if there is a poor response [34].

The aim of this study was to describe the first isolation of an MAA strain in wild otters and to describe its multidrug resistance profile. 

## 2. Case Presentation

### 2.1. Case Description and Postmortem Examination

A female Eurasian otter was found dead on the road in Garešnica, Croatia (S 45°33.743′ E 016°58.267′), due to vehicular trauma and was transported to the Faculty of Veterinary Medicine of the University of Zagreb for postmortem examination. The road where the animal was found is located in the countryside, surrounded by woods near a large fishpond, which was probably the habitat of the otter found. This fishpond is also a habitat for numerous wild birds. There are no houses or farms in the immediate vicinity, but in the wider area, there are two poultry farms where chickens are kept on an “open chicken house” model as well as other private farms that keep chickens free range in the yard.

The animal was in good physical condition but had severe traumatic injury in the area of the head, thorax, pelvis, and limbs, accompanied by severe bleeding and damage of the parenchymal organs, especially the lungs, liver, heart, and kidneys. Additionally, postmortem putrefactive changes were advanced. Apart from the changes due to trauma, the organs, including the lymph nodes, showed no other macroscopic pathological changes that would cause suspicion regarding certain diseases. Histopathologic examination could not be conducted due to the factors of trauma and putrefaction. The animal was examined as part of the bovine tuberculosis eradication and surveillance program prescribed by the Croatian Ministry of Agriculture, the aim of which was the detection of bovine tuberculosis in wild animals.

### 2.2. Bacteriological Examination 

**Figure 1:**
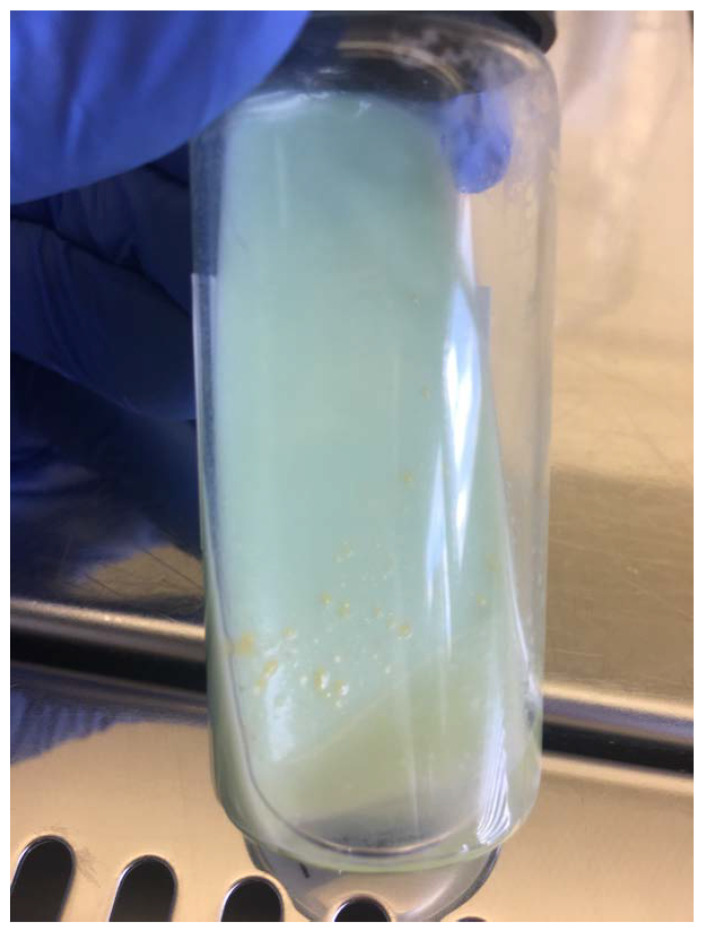
*M. avium* subsp. *avium* colonies isolated from the mesenteric lymph nodes of the otter; morphologically smooth, flat, and transparent, grown on Löwenstein–Jensen slant with glycerol.

The samples of prescapular, submaxillary, and mesenteric lymph nodes were homogenised, concentrated, and decontaminated separately according to the protocol described by Kent and Kubica [35] and inoculated onto standard culture media for *Mycobacterium* species (Löwenstein–Jensen slant with pyruvate, Löwenstein–Jensen slant with glycerol, and Stonebrink slant) and incubated at 37 °C for eight weeks in aerobic conditions. The bacteriological examination was carried out at the Croatian Veterinary Institute, which is the national reference laboratory for bovine tuberculosis. The mycobacterial media are produced in-house according to a prescription adopted from the VISAVET Health Surveillance Centre (Madrid, Spain), the European Union’s reference laboratory for bovine tuberculosis. The media were checked for growth twice a week. Bacterial colonies were observed on Löwenstein–Jensen slant with glycerol for all three samples up to the 20th day of incubation (Figure 1). The grown colonies were smooth, flat, and transparent (SMT), and with further incubation, the colour became increasingly yellow. The presence of acid-fast bacilli was confirmed by Ziehl–Neelsen (ZN) staining in all the samples. Positive colonies were then subcultivated and further identification was carried out using molecular methods. Other organs and tissues were not available for bacteriological examination due to severe trauma and advanced postmortem autolytic changes. 

### 2.3. Molecular Identification

DNA was extracted from the bacterial colonies using a commercial kit (Dneasy Blood & Tissue^®^, Quiagen, Hilden, Germany) according to the manufacturer’s instructions. Further identification was carried out using conventional PCR by the amplification of the DNA sequence encoding the 65kDa antigen common to all mycobacteria using the primers TB1 (5′-GAG-ATCGAG-CTG-GAG-GAT-CC-3′) and TB2 (5′-AGC-TGC-AGC-CCA-AAG-GTG-TT-3′) [36], which amplified a product size of 383 base pairs, confirming that our samples belong to the genus *Mycobacterium*. For further identification, the strain was tested with the commercial DNA strip test Geno Type^®^ Mycobacterium CM (Hain Lifescience, Nehren, Germany) according to the manufacturer’s instructions, which is used for the detection and identification of mycobacteria to the species level and identified our strains as a member of the *Mycobacterium avium* complex. In addition, the isolate was subjected to the PCR amplification of integrated insertion sequence IS*901* using the primers P1 FR300 (5′-CAG-CCA-GCC-GAATGT-CAT-CC-3′) and P2 FR300 (5′-CAA-CTC-GCG-ACA-CGT-TCA-CC-3′) [37]. The amplified product size was 1700 base pairs which proves the presence of the insertion sequence IS*901* and identified our strains as *M. avium* subsp. *avium*.

### 2.4. Antimicrobial Susceptibility Testing 

**Figure 2:**
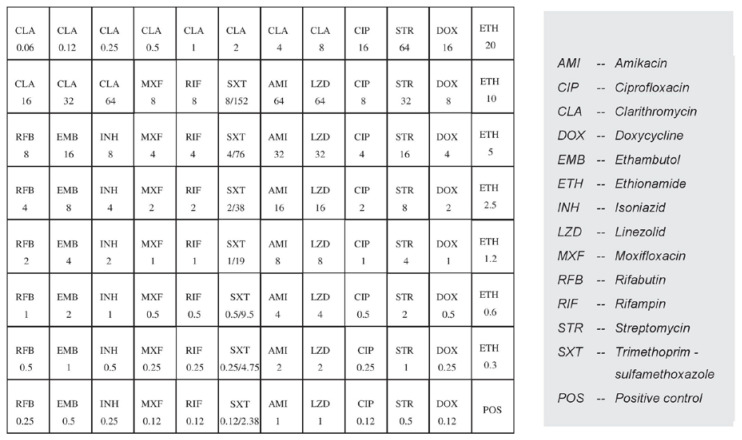
Distribution and concentrations (µg/mL) of the antibiotics on the microtiter Sensititre ™ Myco SLOMYCO AST plate.

The obtained *M. avium* subsp. *avium* isolate was subjected to antimicrobial susceptibility testing (AST) according to the Clinical and Laboratory Standards Institute (CLSI) recommendations. The test was performed according to a standard broth microdilution method for the determination of mycobacterial resistance using the commercial Thermo Scientific™ Sensititre™ Myco SLOMYCO AST Plate kit (Thermo Fisher Scientific, Waltham, MA, USA) [28,29]. The isolate was tested for the following 10 antibiotics that are most commonly used to treat NTM infections in humans: amikacin (AMI), ciprofloxacin (CIP), clarithromycin (CLA), doxycycline (DOX), ethionamide (ETH), isoniazid (INH), linezolid (LZD), moxifloxacin (MXF), streptomycin (STR), and trimethoprim-sulfamethoxazole (SXT). Although they are useful clinically, ethambutol (EMB), rifampin (RIF), and rifabutin (RFB) were not tested since no breakpoint concentrations that distinguish susceptible from resistant strains have been found to date, and because there is no correlation between the MICs found in vitro and the clinical response in patients with MAC infection [29]. The distribution of the tested antibiotics and their concentrations on a microtiter plate are shown in Figure 2.

The bacterial suspension was prepared using a cation-adjusted Mueller–Hinton broth (Thermo Fisher Scientific, Waltham, MA, USA) supplemented with 5% Middlebrook Oleic Albumin Dextrose Catalase Growth Supplement (Sigma-Aldrich, St. Louis, MI, USA), which was incubated at 36 ± 1 °C for seven days. The minimum inhibitory concentration (MIC) was defined as the lowest concentration that inhibits >99% of the mycobacterial growth, or at least 80% of the growth in the case of SXT, and was interpreted in accordance with the CLSI recommendations, which used breakpoints for the MAC testing of CLA, AMI, LZD, and MOX [29]. For the remaining six antibiotics tested, no reference MIC data for MAC are available. Therefore, the MICs of SXT, CIP, and DOX were interpreted according to the CLSI guidelines using breakpoints for *M. kansasii*, whereas STR, INH, and ETH were based on the prior work [38]. The isolate was classified as susceptible, intermediate susceptible, or resistant, as shown in Table 1. Finally, the tested isolate was sensitive to CLA and AMI, while it was resistant to the other eight antibiotics tested (Table 1). Reference strain *M. avium* ATCC 700898 (American Type Culture Collection, Manassas, VA, USA) was used as a positive control.

## 3. Discussion

Following a sharp population decline in the past, the population of otters (*Lutra lutra*) is increasing again in Europe [39]. This is the first study in which a multidrug-resistant strain of *M. avium* subsp. *avium* has been isolated in a wild otter. The fact that no macroscopic lesions of MAC infection were observed could mean that insufficient time has elapsed for their development after infection or that advanced postmortem autolytic changes and trauma factors have masked their presence. However, we should be aware that the detection of NTM species in the sterile areas of the body is usually always clinically significant [40]. MAA was isolated from the prescapular, submaxillary, and mesenteric lymph nodes, indicating an oral route of infection. Otters are predators and eat fish, frogs, and crabs but also other animals such as birds and can even hunt domestic fowl. It frequently changes its habitat, covering a distance of up to 40 km [41]. MAA is considered highly host-specific and is mainly a pathogen of birds, with wild birds considered as one of the reservoirs [13,16]. The area where the otter carcass was found is close to a large fishpond, which is a habitat for numerous wild birds and was probably also the habitat of the otter found. This habitat represents one of the possible sources of MAA infection for the otter. Although there were no poultry in the immediate vicinity of where the carcass was found, this should not be ruled out as a possible source of infection, as otters have a wide range of movement and there were farms with backyard poultry farming in the wider area. Transmission routes other than the oral ingestion of the pathogen should also not be ruled out, as most organs were not suitable for testing. Recent regional studies which described MAA isolates in domestic and wild animals in Croatia [17,18,19,20] correlate with this finding in otters and suggest that wild and domestic animals may be infected with the same species circulating among them and contributing to the spread of such NTMs in the environment. It is also interesting to note the occurrence of such drug-resistant mycobacteria in wild animals, which in principle should not come into contact with antimicrobial agents. In earlier regional studies in Croatia, the resistance of MAA strains isolated from domestic animals, especially cattle, chickens, and ducks, was described. The isolates tested were 100% resistant to moxifloxacin, 83% to linezolid, and 5% to amikacin [23]. This result correlates with our MAA strain which showed resistance to moxifloxacin and linezolid. Another study in Poland reported MAA strains in pet ornamental birds resistant to rifampicin, isoniazid, ethambutol, and ethionamide, and susceptible to streptomycin [42]. Our strain showed resistance to isoniazid, ethionamide, and streptomycin, while rifampicin and ethambutol were not tested. Due to extensive production systems, interactions often occur between domestic and wild species sharing the same environment, increasing the exposure of all the species to these pathogens. In this way, it is possible to transmit the multidrug-resistant MAA pathogen from one species to another. Although direct contact can happen, common habitats play a major role in mediating the transmission of NTM across domestic and wild species [6]. In particular, the subspecies of *M. avium* contaminate the environment through animal excreta, which may serve as a source of infection, but further epidemiological studies are needed to substantiate this claim [5]. Thus, the question of the source of the infection in this otter remains open and it can only give a rough indication of the location of the original source of the infection. The only way to overcome this issue is to collect spatial information on a large sample of animals to determine their infection status [43], as well as their antimicrobial resistance profile.

It is known that NTM infections, including MAC, are not transmitted from human to human but are acquired through the environment [5]. Because of the loss of natural habitats and the growth of cities, human–wildlife interactions are occurring more frequently. Our findings highlight the risk of zoonotic MAC infections, and the established resistance to antibiotics commonly used to treat such infections in humans suggests that wild otters represent a potential reservoir for multidrug-resistant MAC strains. MAC infection is a zoonosis that can be transmitted from environmental reservoirs to domestic animals and humans [44]. 

Species within the MAC are responsible for most human lung diseases caused by NTM worldwide [45,46]. The prevalence of NTM infections in humans has increased in recent years, especially in immunocompromised patients. In the European Union, 99 different NTM species have been described in patients, of which *M. avium*, more precisely MAH, is one of the most common [6,47]. One of the characteristics of the NTM species is a high degree of drug resistance, which makes its treatment much more difficult than that of the *Mycobacterium tuberculosis* complex. To date, there is no standardised antimicrobial therapy for NTM infections [48]. Linezolid and moxifloxacin are among the recommended antibiotics for the treatment of infections caused by *M. avium* spp. in human medicine [26] and are used as the second-line drugs when resistant strains occur to which the first-line drugs (clarithromycin and amikacin) have not responded [28,29], as well as in patients who cannot tolerate macrolide therapy. It is of concern that the isolate of *M. avium* subsp. *avium* in this study showed resistance to linezolid and moxifloxacin, one of the recommended drugs, underlining the great zoonotic potential and leading to challenges in the treatment of such infections in the most susceptible groups. Wildlife researchers as well as National Park rangers and livestock and poultry farmers who are in potential contact should also take additional precautions while handling live or dead specimens. From a veterinary perspective, the recommended therapies for MAA infections in cats and dogs include fluoroquinolones (ciprofloxacin and moxifloxacin) and doxycycline in the case of poorly tolerated rifampicin therapy [30,31], to which our isolate showed resistance. The therapy for pet birds includes isoniazid, ciprofloxacin, streptomycin, and moxifloxacin [16], to which our isolate also showed resistance. Previous studies from Poland and Egypt also reported bird MAA strains resistant to isoniazid, streptomycin, trimethoprim/sulfamethoxazole, and doxycycline, which is consistent with our results, although all the isolates from Poland were sensitive to ethionamide and our isolate was resistant [49,50]

It is already clear that MAA circulates between the environment, wild and domestic animals, and humans, highlighting its potential to cause infections in its hosts. Therefore, it is important to understand the distribution and antimicrobial resistance of mycobacteria in the wild, as information is very deficient. The active surveillance of wildlife reflects what is happening in the environment, which is the main source of infection for humans and coexisting animals [6]. Wild carnivores could serve as an indicator of the prevalence of MAA and other NTM in an area, especially the presence of highly resistant strains.

## 4. Conclusions

This is the first study describing the occurrence of *M. avium* subsp. *avium* in wild otters. While our results highlight the emergence of multidrug-resistant MAC strains among wildlife, several important questions remain unanswered. Additional research on NTMs is needed to gain a deeper comprehension of the ecological niches and the mechanisms involved in the acquisition of organism resistance. The extent to which infected otters are involved in the epidemiology of *M. avium* in other wild or domestic animals remains unknown. It highlights the necessity of additional NTM epidemiology studies in wildlife and their implications for “one health”.

Additional research is necessary to have a deeper comprehension of ecological niches and the mechanisms involved in the acquisition of organism resistance.

## Figures and Tables

**Table 1:** Breakpoints used for *M. avium* subsp. *avium* antimicrobial susceptibility testing with the minimum inhibitory concentration (MIC) values of all drugs included in the panel.

Antimicrobial Agent	MIC (µg/mL) Criteria			MIC Results	Interpretation
	S	I	R		
Clarithromycin ^1^	≤8	16	≥32	2	S
Linezolid ^1^	≤8	16	≥32	32	R
Moxifloxacin ^1^	≤1	2	≥4	>8	R
Amikacin ^1^	≤16	32	≥64	16	S
Streptomycin ^3^	≤16	32	≥64	>64	R
Isoniazid ^3^	-	-	>0.2	>8	R
Trimethoprim/sulfamethoxazole ^2^	≤ 2/38	-	≥ 4/76	8/152	R
Ciprofloxacin ^2^	≤ 1	2	≥4	>16	R
Doxycycline ^2^	≤ 1	2-4	≥8	>16	R
Ethionamide ^3^	-	-	>5	>20	R

^1^ These breakpoints are recommended by the Clinical and Laboratory Standards Institute (CLSI) for *M. avium* complex; ^2^ these breakpoints are recommended by the CLSI for *M. kansasii;* ^3^ the breakpoints are based on Lin, 2022 [38] S—susceptible; I—intermediate susceptible; R—resistant; MIC—minimum inhibitory concentration.

## Data Availability

The original contributions presented in the study are included in the article, further inquiries can be directed to the corresponding author.
